# Proxies for Success: How the Application Process Correlates to PhD Pursuit for a Small Diversity Research Program

**DOI:** 10.1177/2158244017727040

**Published:** 2017-08-30

**Authors:** Dawayne Whittington, Latricia E. Wallace, Cherilynn R. Shadding

**Affiliations:** 1 Strategic Evaluations, Durham, NC, USA; 2 Baylor College of Medicine, Houston, TX, USA; 3 Washington University School of Medicine, St. Louis, MO, USA

**Keywords:** application process, diversity program outcomes, postbaccalaureate research, undergraduate research, STEM

## Abstract

Science, technology, engineering, and mathematics (STEM) diversity research programs seek to make progress in increasing the number of underrepresented students that pursue STEM at the highest degree levels. Yet few programs have outlined their path to help students achieve the STEM PhD. Our program, Opportunities in Genomics Research (OGR), showed significant increases in PhD matriculation over 8 years of National Institutes of Health (NIH) funding. We explored typical measures, which include grade point average (GPA), institution classification, and graduate school ranking, and found that these measures alone do not explain the improved outcomes. We examined changes in the application materials as proxies for commitment to a PhD degree. These data show a significant correlation of desired degree pursuit to increased proxies and proxy type (open- or close-ended questions answered by applicant or referee). We demonstrate that changes in application procedures for diversity research programs correlate to improved program outcomes with statistical significance.

## Introduction

### Why Diversity in STEM Matters

Labor statistics show that the fields of science, technology, engineering, and mathematics (STEM) are the fastest growing, and persons working in these fields earn higher median wages overall compared with other workers (Vilorio, 2014). Since the Sputnik era, funding for programs to increase STEM learning and engagement flourished with the government sponsoring major scientific initiatives, which relied on innovation from the brightest minds (e.g., the Human Genome Project). Continuing massive endeavors from the Precision Medicine Initiative (NIH.gov) to the search for life on Mars will require the continual training of new scholars. Although the STEM fields represent areas of economic and intellectual growth and prosperity for the United States, these fields still reflect the social ills that persist in our society. Underrepresented minorities (URM) represent 31% of the population in the United States, yet comprise only a small portion of bachelor degrees earned in science and engineering fields (~18%; National Science Foundation [NSF], 2013). Specifically, Hispanics (all races) comprise 9%, Blacks/African Americans 9%, and American Indian/Native Americans less than 1%. Only 9% of doctoral degrees (excluding behavioral and social sciences) are earned by URM. Additional data show that URM comprise about 13% of the STEM workforce at the bachelor degree and higher levels, while less than 6% of URM hold full professorships across all U.S. universities (NSF, 2013). While more programs are focusing on disparities at the postdoctoral and faculty levels, the issues experienced at the higher levels of science are a direct result of those that exist at the K-12, undergraduate, and graduate level, from the availability of challenging K-12 STEM curriculum in URM communities to high attrition rates from STEM undergraduate and graduate degree programs (Chen & Weko, 2009; Lichtenberger & George-Jackson, 2013; National Research Council [NRC], 2011).

### Literature Review

#### Benefits of undergraduate research.

Government agencies such as the National Institutes of Health (NIH) and the NSF have addressed this education and workforce gap directly through funding of diversity research and science education programs, which are primarily focused on the recruitment and retention of URM in STEM. These programs vary from summer and academic-year research experiences to innovative STEM curricula in the classroom (Chaplin, Manske, & Cuise, 1998; Mervis, 2010; NRC, 2005; Schultz et al., 2011). Involvement in research has long been considered a key factor in retaining students in STEM (Bauer & Bennett, 2003; Russell, Hancock, & McCullough, 2007). The benefits of undergraduate research include developing a higher interest in science, enhanced science identity and improved self-confidence, scientific skills development, and career path identification (Junge, Quinones, Kakietek, Teodarescu, & Marsteller, 2010; Kardash, 2000; Lopatto, 2004; Seymour, Hunter, Laursen, & Deantoni, 2004). Programs focused on research experiences for URM have demonstrable benefits impacting career trajectories, college persistence, undergraduate grade point average (GPA), and completion of a biology degree (Matsui, Liu, & Kane, 2003; Nagda, Gregerman, Jonides, von Hippel, & Lerner, 1998; Villarejo, Barlow, Kogan, Veazey, & Sweeney, 2008). For example, the Biology Undergraduate Scholars Program at the University of California, Davis, which is comprised primarily of URM, reported that program interventions that included undergraduate research strengthened college persistence to undergraduate STEM degrees, influenced career aspirations, increased STEM graduate degree pursuit, and improved academic performance (Barlow & Villarejo, 2004; Jones, Barlow, & Villarejo, 2010; Villarejo et al., 2008).

Several interventional programs define success as pursuit of graduate degrees in STEM and have demonstrated some influence on this outcome. Bauer and Bennett (2003) surveyed alumni from the University of Delaware to confirm the benefit of undergraduate research experiences. Alumni who participated in the structured undergraduate research program were more likely to pursue a PhD than those who participated in research via other mechanisms or those who did not experience research (67% vs. 21% vs. 12%, respectively). This study was neither focused on URM nor STEM, although 59% of the respondents were STEM majors. One of the first studies to show the influence of undergraduate research on URM terminal degree outcomes demonstrated that URM who participated in research were more likely than their nonresearch counterparts to pursue graduate or professional degrees (MS, PhD, JD, or MD; Hathaway, Nagda, & Gregerman, 2001). They further demonstrated that URM who participated in the Undergraduate Research Opportunity Program were more likely to pursue graduate or professional degrees at similar rates to Whites and Asians in the same program. A study of the national Louis Stokes Alliance Minority Participation (LSAMP) program reported that their participants were more likely to pursue graduate degrees (master’s and PhD) than Whites and Asians and other non-LSAMP URM (Clewell, de Cohen, Tsui, & Deterding, 2006). The authors attributed this success partially to participation in undergraduate research.

Data become more limited when examining PhD outcomes of URM who participated in undergraduate STEM programs during the summer or academic year. Outcomes from the Spend a Summer With a Scientist program at Rice University showed that 62% of the undergraduate participants enrolled in graduate school at the time of the study, but did not specify master’s versus PhD programs (Alexander, Foertsch, Daffinrud, & Tapia, 2000). A longitudinal study by Foertsch, Alexander, and Pernberthy (1997) showed that 52% of URM participants in a summer research program pursued graduate school that included but is not limited to master’s and PhD programs (Foertsch et al., 1997). When restricting to PhD, Rivera and Murray (2014) showed the positive influence of program components, such as mentorship, graduate record exam (GRE) workshops, and graduate school preparation, on URM in choosing a career in STEM, where 26% of program alumni matriculated into PhD programs in STEM (Rivera & Murray, 2014).

The well-known Meyerhoff Scholars program at the University of Maryland Baltimore County provides a closer assessment of interventions on URM PhD pursuit. Early studies of the Meyerhoff program show that participants, largely African American, are more likely to enroll in graduate programs compared with students who declined the program and the students who attended the institution prior to the establishment of the program (Maton, Hrabowski, & Schmitt, 2000). Additional studies demonstrated that students pursuing a PhD entered college with higher research excitement than those who pursued MD or no graduate degree (Maton, Sto Domingo, Stolle-McAllister, Zimmerman, & Hrabowski, 2009). They also found that summer research increases the chance of URM students enrolling in a STEM PhD program and that multiple experiences have a cumulative effect (Pender, Marcotte, Sto Domingo, & Maton, 2010). The outcomes of the Meyerhoff program are also linked to PhD completion. Maton et al. (2016) showed that participation in Meyerhoff for URM increased the likelihood of completing a PhD, where African American Meyerhoff students were more likely to complete a PhD than comparable students who declined the program (Maton et al., 2016).

Although there is much data regarding undergraduate research and its benefit to URM, the data detailing the effects of diversity research programs on URM PhD pursuit are limited to a few programs. Thus, deficits remain in our understanding of how these diversity research programs affect PhD pursuit in STEM by underrepresented groups.

#### Benefits of postbaccalaureate (postbacc) research.

Even after the baccalaureate is completed, many URM students lag behind their non-URM counterparts who, in addition to having more advantages at the start, continue to add to their skills and opportunities over the course of their undergraduate and graduate training (McGee, Saran, & Krulwich, 2012). To address remaining gaps, postbacc research programs were created to provide additional experiences for recent URM college graduates. Postbacc programs that focus on graduate education as a successful outcome exist in many institutions. These programs provide a subset of experiences that include research, course-work, and GRE preparation to better equip students for PhD or MD/PhD program matriculation. The most notable postbacc programs are those funded by the National Institute of General Medical Sciences (NIGMS): Postbaccalaureate Research Education Programs (PREP) established in 2000. The limited published data on benefits and outcomes of postbacc programs come mostly from the PREP programs. McGee et al. (2012) showed that over one half of the PREP participants at Mount Sinai School of Medicine entered PhD or MD/PhD degree programs where the PREP program focused on developing talent rather than accepting students expected to succeed (McGee et al., 2012). A report from the NIGMS showed that the national PhD matriculation rate of PREP alumni was 65% and the PhD completion rate was 63%, indicating that PREP participants likely contribute to the diversity of the STEM workforce (A. Hall, Mann, & Bender, 2015).

PREP programs are not merely for students who have committed to the PhD path. Gazley et al. (2014) demonstrated this by categorizing five patterns of PREP participants: principal investigator (PI) aspirants, credential seekers, interest testers, path builders, and discipline changers, where some participants sought the PREP for their first research experience (interest testers) or more experience (PI aspirants), for guidance on the PhD career path (path builders), or to fill in academic gaps of non-STEM majors (discipline changers; Gazley et al., 2014). A recent study by this group showed that over 85% of these same PREP scholars pursued a PhD or MD/PhD with all patterns of PREP participants represented in these outcomes (Remich, Naffziger-Hirsch, Gazley, & McGee, 2016). Another study from the University of North Carolina at Chapel Hill showed remarkable outcomes where over 90% of PREP participants entered PhD programs with a 95% retention rate. The program components most highly rated by participants included the research experiences as well as mentoring by PhD-trained program staff (J. D. Hall et al., 2016).

It is generally agreed that research experiences at the undergraduate and/or postbacc level have positive effects on URM pursuit of PhDs in STEM. Some of these studies have begun to explore why and how these effects occur. Most of the reports so far indicate that the program components (e.g., mentorship, research skills, etc.) are important, while others indicate that self-identity and self-efficacy are key and may mediate the effects of the program components (J. D. Hall et al., 2016; Maton et al., 2016; McGee & Keller, 2007; Remich et al., 2016). Thus, more research is essential to understand the impacts of diversity research programs on URM PhD pursuit and completion.

The McDonnell Genome Institute at Washington University in St. Louis (WU) hosts the Opportunities in Genomics Research (OGR) programs, which are focused on URM students with the key goal of increasing the number of students who pursue PhDs. OGR is comprised of a summer research program and a distinct 1-year postbacc program. We have previously shown results from our summer program that demonstrated that lower cost recruitment methods (i.e., email, referrals) were equally as effective as higher cost methods (i.e., conferences) in recruiting summer students who pursue PhDs (Shadding, Whittington, Wallace, Wandu, & Wilson, 2016). In the present study, we focus on both our summer and postbacc program collectively. During the 8 years of funding for the OGR program, we noticed a more than twofold increase in PhD outcomes from our first cycle (2007–2011) to the second cycle (2012–2015) for both programs combined.

The goal of this study is to investigate parameters that account for this increase. We examined two possible causes. First, the credentials of the students in the program may have improved over time. We examined measures of quality, including GPA, undergraduate institution, Carnegie classification, and ranking of graduate institution, to determine if these measures accounted for our improved outcomes across funding cycles. Second, we explored our application process, which is an understudied parameter. Our application materials increased the number of questions that served as proxies, for PhD commitment for each major component of the application process.

Most diversity research programs have a thorough selection process. While some programs, such as the Meyerhoff program, have evaluated their selection criteria (e.g., SAT scores and high school GPA) as predictors of desired outcomes, we have not observed in the literature how well an application can gauge an applicant’s genuine interest in PhD pursuit and whether the outcomes confirm this.

We explore the relatedness of the application process to PhD pursuit for URM in diversity research programs. We suggest that these results will be beneficial for new programs that are establishing their metrics for success or for older programs seeking to enhance their success. This work may also have implications for funding agencies as they evaluate and advise programs on how to improve outcomes.

## Method

We asked the following research questions to explain the significant improvement in student enrollment in PhD programs in the second cycle:

**Research Question 1:** Did the participants who enrolled in PhD programs have stronger academic credentials?
**Research Question 2:** Did changes in the application process enhance selection of participants who enrolled in PhD programs?

### The OGR Program

OGR programs were established in 2007 through the NHGRI (National Human Genome Research Institute) Diversity Action Plan at the McDonnell Genome Institute at WU in St. Louis. OGR is dedicated to the recruitment and retention of URM students, which directly addresses national concerns of disparity in degree attainment in the sciences. The main goal of OGR is to increase the number of URM students who pursue PhD degrees in genomics and related fields. Currently, OGR has two programs to accomplish this goal: (a) Undergraduate Scholars (OGR-US, established 2007) and (b) Extensive Study (OGR-ES, established 2008). OGR-US is an 8-week summer program and OGR-ES is a 1-year, postbacc program for recent college graduates. In both programs, students conduct independent research with investigators at WU and participate in activities directed toward graduate school readiness and STEM career success. These activities include GRE preparation, a graduate school preparation course, journal club, presentation skills workshop, and individual advising. While both programs share similar activities, only a few students, *n* = 6, transitioned from the summer program to the postbacc program from 2007 to 2015.

### Data Collection

To address questions of how the OGR programs evolved, we assessed the degree outcomes of OGR alumni. We present data for OGR-US from 2007 to 2014 (*n* = 67) and OGR-ES from 2007 to 2015 (*n* = 21), for a total of 88 participants. During the reported cycles, matriculants of the OGR-US program were allowed to return for subsequent summers and/or could enter OGR-ES noncompetitively with demonstrated progress toward the goals of the programs. We present collective data on our OGR programs, comparing grant cycles of NHGRI funding for OGR, where data reflect unique students only; students who return to our programs are only counted once. It is worth noting that the OGR-US alumni (summer) students who transitioned to our program are only accounted for in the summer data; thus, the 21 postbacc participants are those with no prior exposure to the OGR program.

The baseline data were extracted from applications to the program. All data are reported in aggregate by cycle. Demographic information such as gender, race, and ethnicity were voluntary and self-reported on the OGR application. OGR students’ long-term career outcomes, including career path data, were collected from participants and stored in iBioSketch.com, an Internet-based career tracking tool designed by Strategic Evaluations, Inc., our external evaluation team. These career outcomes were verified through at least two other sources: (a) study leaders’ follow-up communication with alumni and their research mentors, and (b) queries submitted to the National Student Clearinghouse. OGR participants were asked to initiate and update their profiles in iBioSketch annually, while formal surveys were given at least biannually. We supplemented this information with informal tracking methods (phone calls, social media, emails, etc.). The reported outcome data indicate a student’s career status as of June 2016 or the last reporting of the student.

Quality metrics were assessed in several ways. First, undergraduate institutions were classified using basic Carnegie classification (Carnegie Foundation for the Advancement of Teaching, 2012) and condensed into the following major categories—associates (includes private and public), bachelors (includes baccalaureate arts and science, diverse fields, and baccalaureate/associates), master’s (small, medium, and large), doctoral (doctoral/research universities), and research (research university with high or very high activity)—and compared by cycle. Undergraduate institutions were also classified as a minority-serving institution (MSI) based on data from the Department of Education listings of minority institutions and Excelencia in Education (Edexcelencia.org, 2014). The following categories for MSIs were used: HBCUs (historically Black colleges and universities), HSIs (Hispanic serving institutions), or non-MSIs (majority institutions or primarily White institutions). To determine student quality metrics, we compared the entering GPAs (STEM and overall) of participants, by cycle, which were reported on the application and verified by official transcripts.

To assess the quality of the institution where the students matriculated for doctorate degrees, we used the data from iBioSketch and annual surveys and recorded the ranking of the institution using U.S. News and World Report (http://grad-schools.usnews.rankingsandreviews.com/).

We conducted an item analysis, to determine which questions in our application materials were likely to yield responses to help us determine the applicants’ commitment to the PhD path. Although our materials have changed and there are some distinctions in the programs, there have been three consistent steps to the process: application, faculty recommendations, and interview with the program director for a subset of applicants whose print materials are highly competitive, thus every student selected for OGR goes through the interview step. At each level of our process, all applicants are asked the same questions in number and content. From these total questions we measured how many probed for PhD interest and labeled these as proxies. In conducting the item analysis, we tabulated the number of questions from each step that could serve as proxies for interest in PhD pursuit and measured the changes in the number of proxies from years 2007 to 2015. We provide a list of the questions that we identified as proxies from our application, faculty recommendations, and interview protocol for 2008 and 2015 to show the growth in proxies from Cycle 1 to Cycle 2 (Supplemental Material).

### Statistical Analysis

*IBM SPSS Statistics Version 21* was used to compute descriptive statistics and test for statistical significance. Independentsample chi-square tests were used to test the distributions for categorical response variables, which included differences between demographic variables and variables serving as proxies likely to yield responses for our team to determine the quality of students selected across the two funding cycles. When appropriate, the crosstab function within SPSS was used to determine if column proportions were significantly different. In these cases, *z* values were computed. To protect against Type I error as a result of the multiple comparisons, the Bonferroni technique was used to adjust the alpha values to make the criteria smaller and therefore more stringent (Huck, 2004). To determine whether the means for our scale variables (overall GPAs and STEM GPAs) were equal across our independent categories, one-way ANOVAs were conducted (Huck, 2004). To determine the strength of correlation between application changes and degree pursuits, applications between 2007 and 2015 were analyzed in relation to the number of items they included that served as proxies for interest in PhD. Proxies were tallied based on whether they were open-ended or close-ended, and whether they were asked of the student or the student’s referees. In addition, degree pursuits were coded into one of four categories and given ordinal values. The Kendall rank correlation coefficient was used to measure the ordinal association between the number of application proxies included to gauge students’ interest and the degree pursuit outcome variable.

## Results

### OGR Demographics

Both OGR programs are small and share similar components. One major difference, other than program length, is that we only accept students who have at least one semester or equivalent of research experience for the Extensive Study program. Due to the similarities and the small numbers of participants, we combined data from both to determine any meaningful change in outcome. We compared data for the two cycles of funding for the OGR programs 2007–2011 (first cycle) and 2012–2015 (second cycle).

Table 1 shows the baseline demographics of our programs. The focus of OGR is underrepresented students (initially URM but then expanded in 2011 to underrepresented which includes first generation, low socioeconomic status, etc.). While the racial and ethnic demographics of OGR diversified in the second cycle, most program participants identified as Black or non-White Hispanic. These groups were in the “majority” for both cycles of OGR’s existence; however, the percentage of Hispanics remained relatively stable while the percentage of Blacks entering the program decreased over the cycles (55% vs. 49%). We also noted a significant increase in the percentage of students identifying as Caucasian, which includes both Hispanic and non-Hispanic Whites (2% vs. 13%; χ^2^ = 13.266, *p* = .039). We used the federal classifications for race and ethnicity in our application for applicants to voluntarily submit their ethnicity and race; thus, a person who self-identifies as Hispanic (ethnicity) may also identify as Caucasian (race). Overall, our programs were relatively gender balanced. In the first cycle, the percentage of females trended higher than males, and in the second cycle, this trend reversed (χ^2^ = 0.696; *p* = .404).

### OGR Degree Outcomes

The OGR programs are comprehensive and have provided research experience and supplemental activities to an academically diverse group of students with varied interests in STEM careers. However, the ultimate goal of OGR is to encourage students to pursue the STEM PhD. We compared our cycles of NHGRI funding to see if there was any improvement in this outcome over time. We grouped the key outcomes for our alumni as PhD, Postbacc/master’s, STEM professional degree (MD, DDS, PharmD), and other. The outcomes represent what they were pursuing or the degree obtained at the time of the 2015 alumni survey, and updates to iBioSketch and do not indicate multiple outcomes. Our data indicate that participants pursuing or completing the PhD significantly increased from the first cycle to the second cycle (27% to 59%), while those pursuing a STEM professional degree significantly decreased from the first to second cycle (22% vs. 5%, χ^2^ = 11.137, *p* = .011; Table 2). There was no significant change in the remaining categories, but there was a clear shift. The PhD was the most common degree outcome in the second cycle at 59%, and “Other” was the most common outcome in the first cycle at 43%. We examined the demographics of PhD matriculants, mostly non-Hispanic Blacks and non-White Hispanics, which make up the largest race/ethnic groups of OGR participants. Across both cycles, the numbers of each group pursuing a PhD is virtually the same (*n* = 16 vs. 18; Table 2). Overall, the percentage of Hispanics pursuing PhD in our sample is higher than that of Blacks, although the percentage of Hispanic PhD matriculants changed, but not significantly (40%—Cycle 1 to 67%—Cycle 2). However, Black PhD matriculants significantly increased from 19% to 58%, doubling the number of Black PhD matriculants (*n* = 5 vs. 11) over the two cycles, in spite of their slight decrease in representation in the program (55% vs. 49%).

Thus, we made major strides to reaching our desired goals of significantly increasing PhD matriculation.

### Measures of Outcomes

We certainly believe that with experience, and acting on results from our external evaluation, our program improved over time. But, we wanted to identify key determinants in these changing outcomes, so we investigated measures of quality as possible answers to why we saw the improvement to PhD matriculation.

One variable we examined was students’ GPA during the application process. We analyzed the overall and STEM GPAs of all participants to determine if there was a more competitive group academically in the second cycle. The overall GPA over both cycles was statistically the same (3.45 vs. 3.41), with an overall GPA that trended slightly higher in the first cycle (*F* = 0.273, *p* = .603), while the STEM GPA trended higher in the second cycle (3.29 vs. 3.32), but was not significant (*F* = 0.089, *p* = .766; Table 3).

We also compared the classification of their undergraduate institutions across the two funding cycles. We categorized the institutions of participants by MSI status and by Carnegie classification. In the first cycle, most participants attended an MSI (HBCU: 37% and HSI: 33%), where 31% attended a non-MSI (Table 4). In the second cycle, a slightly larger percentage of our participants attended undergraduate at non-MSI institutions at 51% (Table 4). In both cycles, the majority of students came from institutions classified by Carnegie as research institutions. When we considered those that pursued PhD only, we see, collectively, the majority of PhD matriculants came from non-MSIs (*n* = 15) and from research institutions (*n* = 15). The observed increase in PhD outcomes in the second cycle, however, was likely driven by non-MSIs but not by research institutions, where there was a more even distribution across Carnegie classifications in the second cycle compared with 62% of PhD matriculants, attending undergraduate at research institutions in the first cycle.

We considered the rankings of institutions that participants attended for their PhD by cycle to determine if the improvement in PhD outcomes may have been accounted for by matriculation into less selective PhD programs. Analyzing rankings by U.S. News and World Report of PhD programs, we saw a small but not a significant increase in rankings in the second cycle from a mean ranking of schools attended, 44 versus 29 (*F* = 1.441, *p* = .239; Table 5). Although not significant, we believe this trend was driven by Black PhD matriculants, where the mean ranking significantly improved for this group from 58 to 20 (*F* = 7.537, *p* = .017) and remained stable for Hispanic PhD matriculants (*F* = 0.005, *p* = .946; Table 5). This overall improved ranking was likely driven by a significant, fivefold, increase in OGR alumni accepted to WU from Cycle 1 to Cycle 2 (*n* = 2 vs. 11, χ^2^ = 10.037, *p* = .002; Table 6).

### Evolution of the OGR Application

To further explore the improved outcomes we observed in Cycle 2 of funding, we also examined our application process. Any selection process is inherently designed to obtain the best talent or to query which candidates are most likely to achieve program or institutional goals. Through evaluations of our program, we made some changes to our program accordingly but we also made changes to the application process for the OGR programs. In the beginning of OGR, our application process was designed to capture students who were interested in research and would consider a PhD among their postbacc options. In later years, we tailored our materials to capture participants with demonstrated greater interest and commitment to the PhD path.

**Figure 1.**
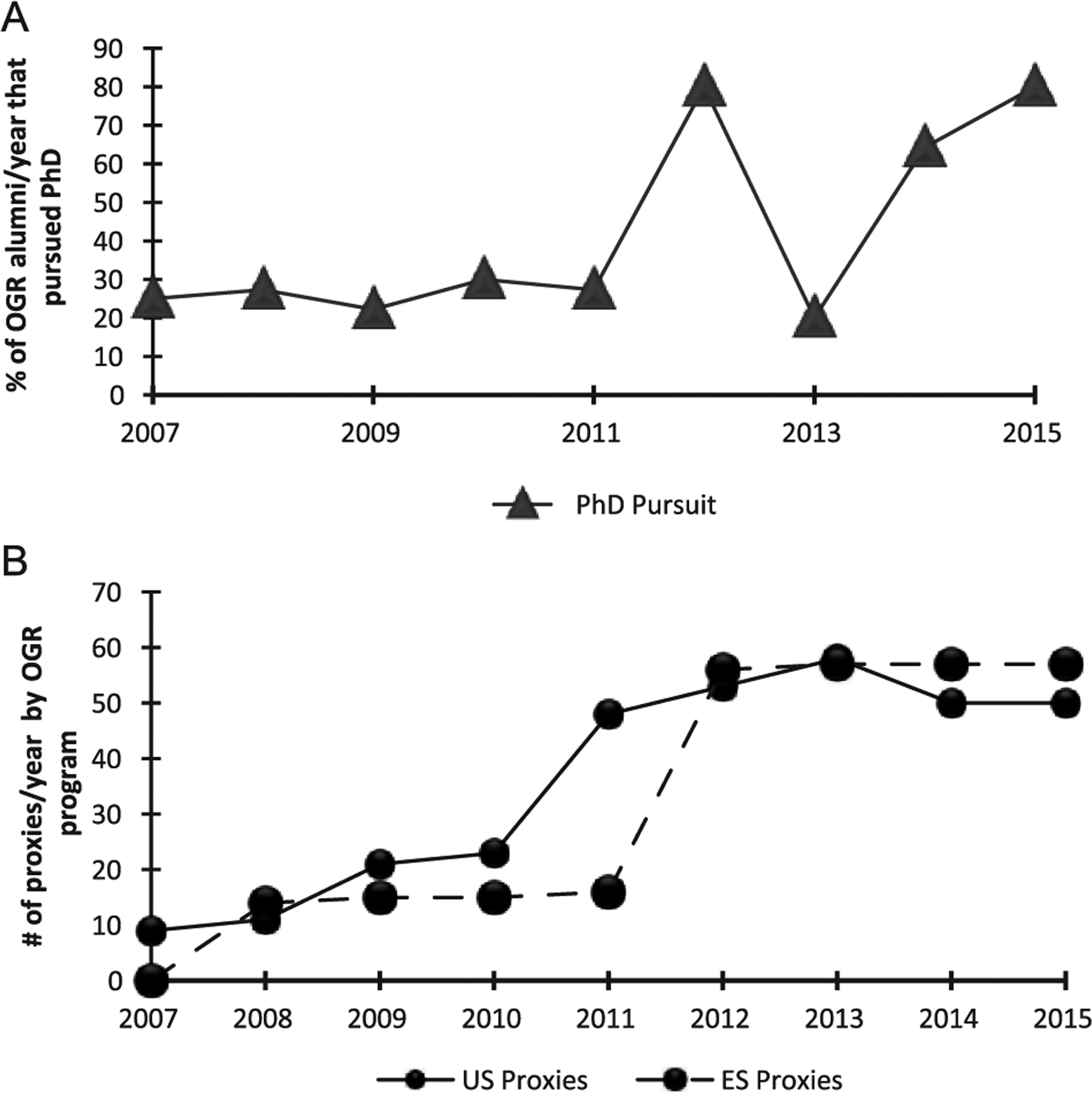
Increases in percentage of PhDs pursued and application proxies for pursuit: (a) the percent of PhDs pursued by OGR alumni by year of their participation in OGR was calculated and (b) the number of application proxies per year was tallied for each year of the program. *Note*. OGR = Opportunities in Genomics Research.

To determine if changes in the application process corresponded to the changes in degree pursuit, we examined all of our applications for each program for each year and quantified the total number of questions that were proxies for interest in pursuing a PhD. For example, in the earlier years of OGR, we asked applicants if they planned to enter a professional degree program and asked them to indicate their program of interest with choices provided (e.g., PhD, MD/PhD, MD, DDS, master’s, and other). In later years, we expanded this by asking them to indicate if they planned to enter a professional degree program or a graduate degree program or if they applied for admission to either program type. We also asked them to indicate any standardized tests they planned to take (e.g., GRE, MCAT, PCAT, DAT). These are just some examples of proxies that helped us determine their level of interest in PhD as well as consistency in their responses (Supplemental Table). Through this item-by-item analysis, we compared the number of proxies with the percentage of participants who applied to PhD and who entered PhD programs by cohort. We emphasize that all applicants saw all questions and the total number of questions was the same for that specific year in which students applied, but we examined if the balance of questions for PhD interests changed over time. Our data show that the number of proxies increased by fourfold (OGR-ES) and sixfold (OGR-US) at its peak in 2013 from the beginning of the programs (Figure 1). With some exceptions, we also saw an overall increase in the percentage of PhD applicants and matriculants in the years where the proxies for both programs were the highest. Beginning in 2011, we made robust important changes to our application for the OGR-US program and then in 2012 for both programs, which corresponds to one of our highest peaks of PhD pursuit.

**Figure 2.**
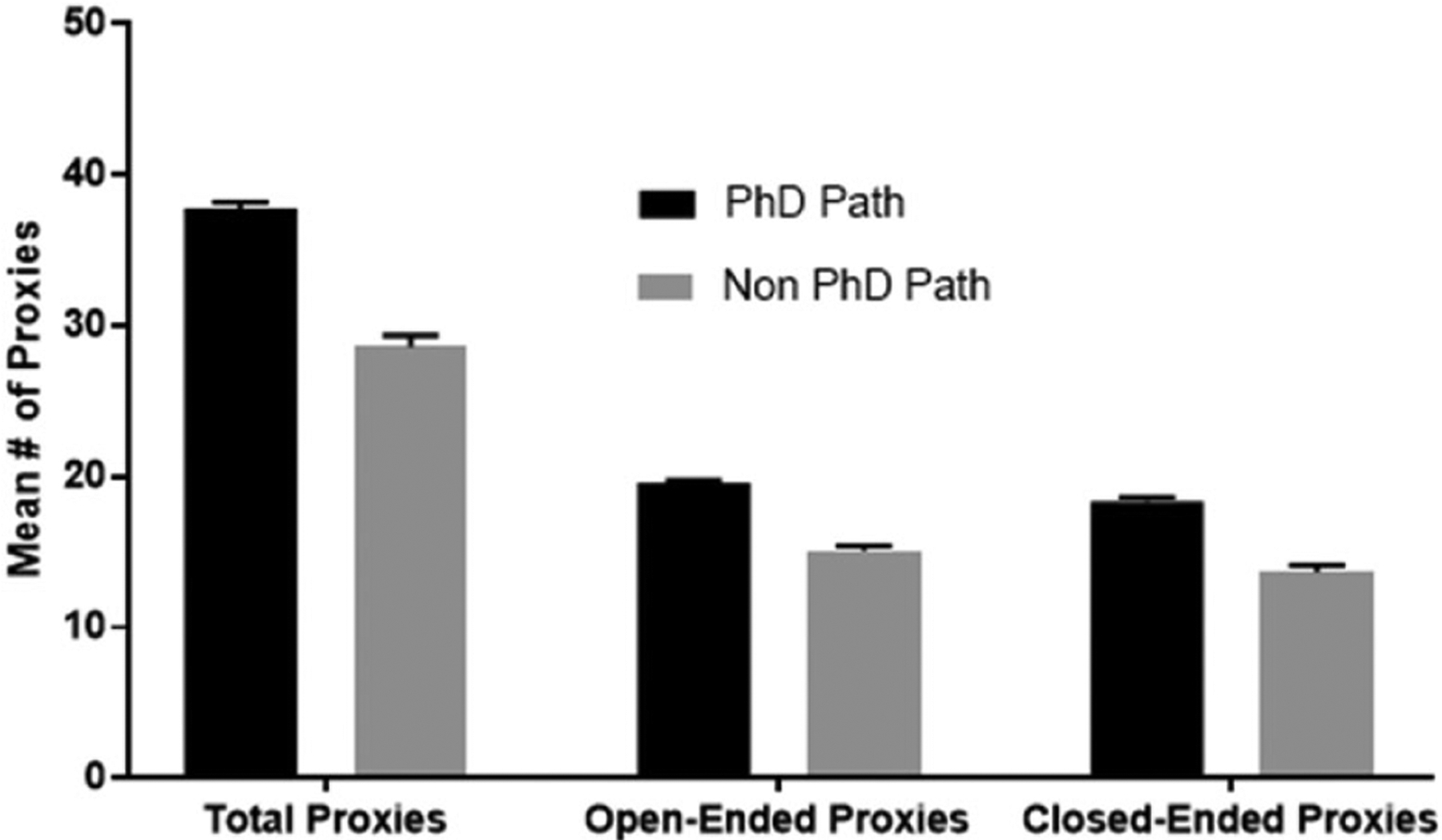
Analysis of student proxies. *Note*. An ANOVA was used to test for differences among PhD and non-PhD path matriculant for total, open-ended, and close-ended proxies students were asked to complete. The *p* values for comparisons across the two groups were .012, .005, and .026, respectively. Eta squared values were .071, .088 and .057, respectively.

**Figure 3.**
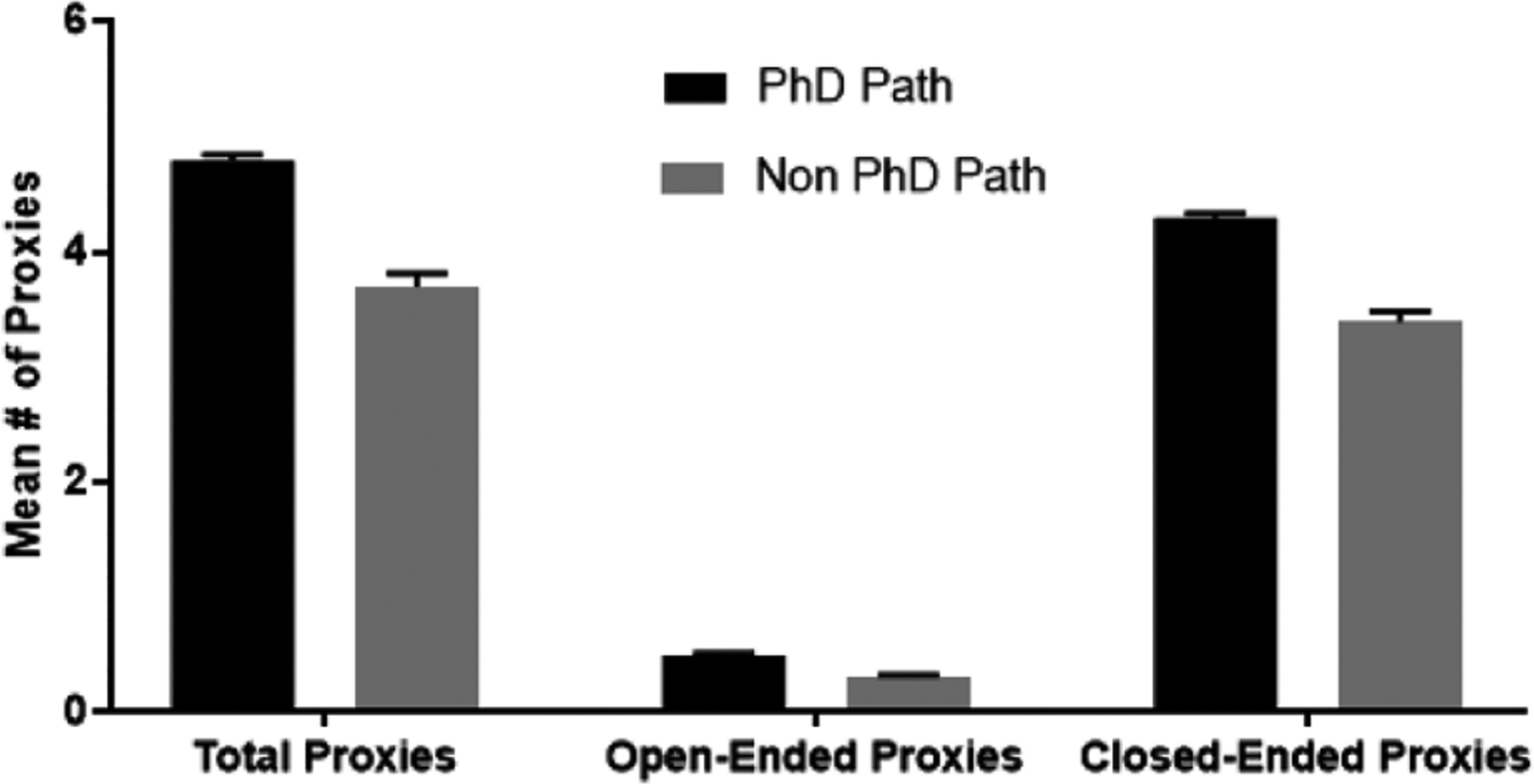
Analysis of referee proxies. *Note*. An ANOVA was used to test for differences among PhD and non-PhD path matriculants for total, open-ended, and close-ended proxies referees were asked to complete. The *p* values for comparisons across the two groups were .014, .195, and .017, respectively. Eta squared values were .068, .019 and .064, respectively.

We further investigated the proxies and categorized these questions as open-ended (example proxy—“what steps have you taken to pursue your career of interest?”) or close-ended (example proxy—“have you presented at a biomedical conference?”). We also categorized the questions by who was required to answer the question, the applicant or the referee (Supplemental Table). To see if there was a difference in degree pursuit and the types of questions that were asked, we performed an ANOVA and considered alumni as either on the PhD path (alumni who enrolled/completed PhDs or MD/PhDs, or currently enrolled in master’s or postbacc) versus non-PhD path (enrolled/completed STEM professional degrees such as MD, DDS, allied health, or non-advanced STEM degree pursuits). We see that students on the PhD path, on average, were asked to respond to significantly more proxies than their peers on the non-PhD path (Figure 2). For example, students on the PhD path were asked to respond to 38 total proxies, where 20 were open-ended and 18 close-ended. Their peers on the non-PhD path were asked to respond to 29 total proxies, 15 open-ended and 14 close-ended (*F* = 6.550, *p* = .012; *F* = 8.287, *p* = .005; *F* = 5.160, *p* = .026, respectively; Figure 2). Similarly, referees were asked to respond to significantly more proxies (total and close-ended) related to the PhD path versus non-PhD path (*F* = 6.308, *p* = .014; *F* = 5.894, *p* = .017, respectively; Figure 3).

**Figure 4.**
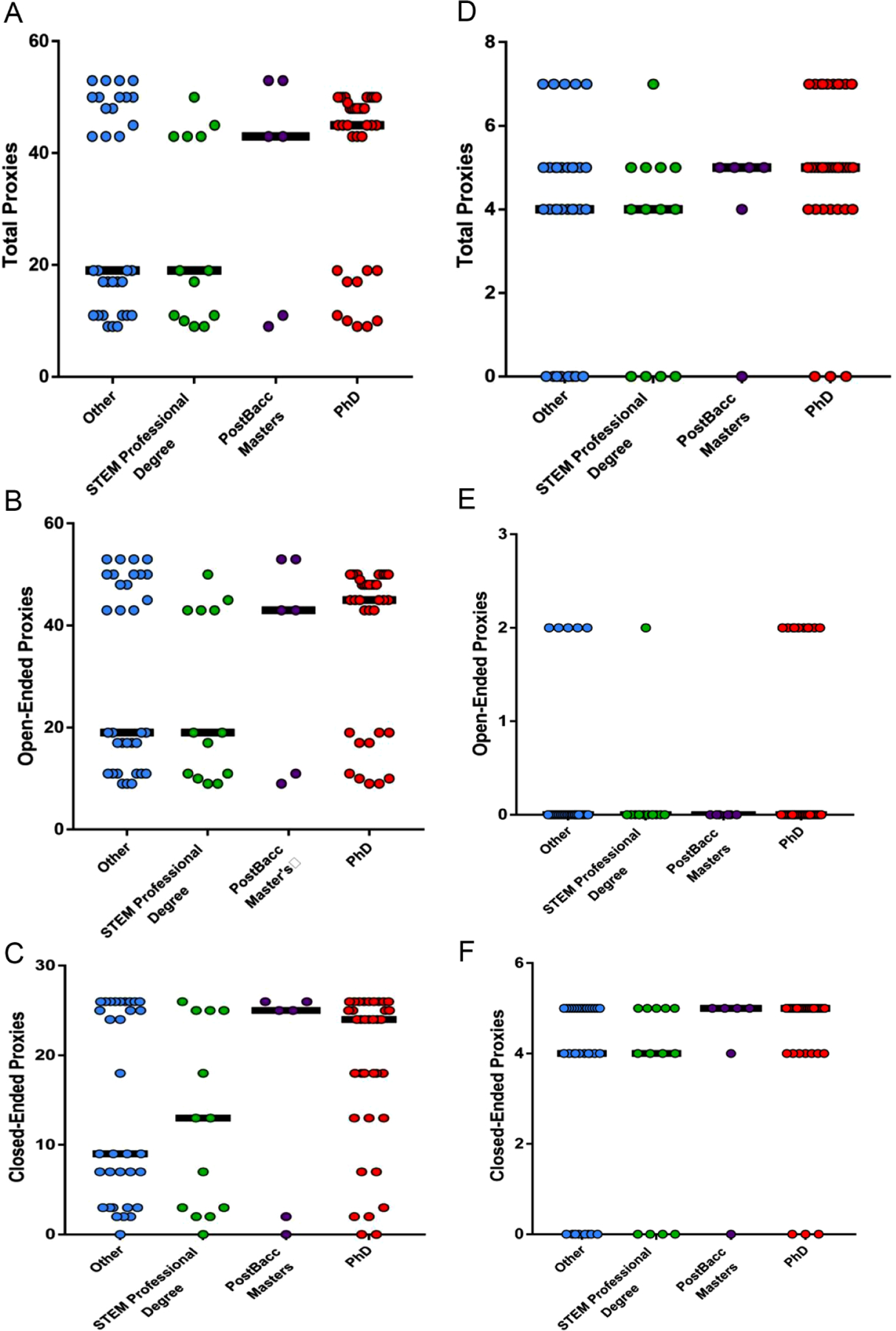
OGR student (a, b, c) and referee (d, e, f) application proxies in relation to degree pursuit outcomes. *Note*. Each circle represents one student, and the black bars represent the median for each population. A Kendall tau correlation test was used to determine association between proxies and degree pursuits. The most desirable degree pursuit outcome, pursuit of a PhD or MD/PhD, was assigned the highest value of 4. Pursuit of a postbaccalaureate certificate or STEM Master’s degree was assigned a 3, while pursuit of a STEM professional degree was assigned a 2. Students not pursuing any advanced degree in STEM were assigned a 1. The *p* and *r* values for correlations for the data are (a) *p* = .121, *r* = .134; (b) *p* = .029, *r* = .195; (c) *p* = .271, *r* = .096; (d) *p* = .014, *r* = .227; (e) *p* = .137, *r* = .172; and (f) *p* = .232, *r* = .016. OGR = Opportunities in Genomics Research; STEM = science, technology, engineering, and mathematics.

We wanted to determine if there was a significant correlation between question type and desirable degree pursuits. Here, we categorized degree types as with earlier data (PhD, master’s/postbacc, STEM professional degree, and other). The most desirable degree pursuit outcome, pursuit of a PhD or MD/PhD, was assigned the highest value of 4. Pursuit of a postbacc certificate or a STEM master’s degree was assigned a 3, while pursuit of a STEM professional degree was assigned a 2. Students not pursuing any advanced degree in STEM were assigned a 1. We see that there is correlation with our desirable degree outcome variable and question type and respondent type. The desirable degree outcome variable was significantly correlated with the number of open-ended items students were required to complete on their application (Figure 4b), but not with total proxies and close-ended items (Figure 4a and 4c). Desirable degree pursuits were also significantly correlated with the number of total and close-ended proxies referees were asked to complete (Figure 4d and 4f). These results suggest that who answers the questions, referee or applicant, and the question format, open- or close-ended, may matter to capturing applicants who will pursue desired outcomes for a diversity research program. Collectively, the data from the ANOVA and correlation (Figures 2–4) suggest that our application improved in capturing students who were committed to the PhD path.

## Discussion

The OGR programs have operated since 2007 with two cycles of funding from the NHGRI. With the key goal of increasing URM who pursue PhDs, the percentage and number of OGR alumni pursuing PhDs increased significantly over these two cycles from 27% to 59%, representing a twofold increase. We investigated this improvement by addressing two questions: whether we recruited academically stronger students and if the increase in outcomes correlates with changes in our application process. We suggest that proxies for commitment to pursuing a PhD degree in the application materials can facilitate an increase in outcomes for diversity research programs.

Clearly, the students must have at least minimal qualifications to be successfully admitted into a PhD program. When we examined GPAs, we saw that participants did not have significantly different GPAs from Cycle 1 to Cycle 2. We further validated this by the Carnegie classification of their undergraduate institutions, and do not observe a significant increase in alumni who attended research institutions, which are the largest producers of undergraduate alumni who pursue PhDs as well as the largest producer of PhDs overall (Fiegener & Proudfoot, 2013). Yet nationally, the baccalaureate origins of URM PhD recipients are more modest and diverse. Black students are the driver of the increase in PhD matriculation over the cycles in our sample, although Hispanic students had the highest PhD matriculation overall (51% vs. 35%). The baccalaureate origins of our Black PhD matriculants were varied where a little more than one half of our alumni hailed from HBCUs of different Carnegie classifications. This corresponds to national data where the baccalaureate schools with the most Black alumni who earned STEM PhDs are mostly HBCUs and are not exclusively research institutions (Bonner, Alfred, Lewis, Nave, & Frizell, 2009; Fiegener & Proudfoot, 2013).

We considered whether students entered less competitive PhD programs, and found a slight improvement in the overall ranking of PhD programs from Cycle 1 to Cycle 2. We also note that these rankings do not fully capture the prestige of some of the institutions where OGR alumni were accepted or matriculated. Due to the specific nature of PhD programs, largely based on field of study, such rankings can be higher or lower than the national overall rankings for that institution. We have had students accepted to Harvard, Berkeley, and Yale and matriculated at Princeton and Johns Hopkins. The improved outcomes we saw in Cycle 2 also correspond to a significantly higher acceptance of OGR alumni at our own institution, WU in St. Louis, at a fivefold increase in acceptance, further validating that something beyond institutional and student quality was responsible for the improvement.

A common challenge for PhD focused diversity research programs is establishing an application process that distinguishes applicants who are truly interested in the PhD versus those seeking research experience for MD applications or other health related programs. We made major changes to our application materials in 2011 and 2012 for both programs. For example, in 2012, we switched to an application form for the OGR-ES program for applicants to list their basic data, research experiences, future career choice, and so on, where previously we allowed them to apply with a cover letter, CV, and letters of recommendation, similar to a postdoctoral fellowship. This automatically increased the measurable proxies for interest in PhD pursuit. For OGR-US participants, there was always a form, but in 2011, we added questions that we believe aided in our evaluation of a candidate’s commitment to pursuing a PhD, for example, having them indicate the types of programs they plan to apply to (graduate vs. professional degree programs) in addition to them indicating their degree of interest (PhD, MD, DDS, etc.).

Our data trends show that as proxies for interest in PhD increased, our desired outcomes increased overall. There were some exceptions, such as in 2013, after the changes were solidified for both programs, we saw a decline in PhD pursuit to Cycle 1 levels. This may be due to a combination of effects: students who applied to PhD but were not accepted or were provided with more costly options to enter (e.g., earning a master’s then PhD), students opted to take some time off before graduate school, and we accepted students who were on the MD versus PhD “fence”, as well as losing PhD committed applicants to other programs.

Our selection process involves an application form that captures basic data and research experience, short answer questions about career interest, and two (OGR-US) or three (OGR-ES) recommendation forms from faculty. After this first screen, students are selected for phone interviews that have just over 20 questions with probes that identify interest, knowledge, professionalism, career plans, motivations, influences, strengths, and weaknesses. As such, our application materials consist of both open-ended and close-ended questions that assess cognitive and/or quantitative measures (GPA, courses taken) and noncognitive and/or qualitative factors (career plans, motivations/influences for career choice, interpersonal, and written skills). This combination of factors allows us to view the applicants comprehensively. Using ANOVA, we determined if there was a difference between those pursuing the PhD path and non-PhD path and saw that, over time, those who pursued the PhD path and their referees were asked more questions that were proxies for interest in this path, both open- and close-ended. We investigated further and found correlation of question type and respondent type to likelihood of pursuit of desirable degree path. We found that the total proxies and number of open- and close-ended questions asked of the applicant and the number of total proxies and close-ended questions asked of the referee correlated with the higher likelihood of pursuit of the desired PhD degree path. Although graduate and medical schools are relying more on noncognitive factors and utilizing comprehensive review to assess candidates, we believe, however, these are the first such data for diversity research programs that are training students for terminal degree pursuit in STEM.

Historically, our program has not focused on the cognitive factors as the key determinant of program entry but rather on student potential. As we increased proxies for PhD commitment on the application, we note that most of the proxies are qualitative (e.g., What degree do you plan to obtain? What steps have you taken to pursue a career in STEM?). The literature regarding question types in application materials and program outcomes for diversity research programs at most is extremely limited. Programs have noted their selection criteria and how this may relate to the outcomes they observed (Jones et al., 2010; Maton et al., 2009). But we have not seen in the literature how qualitative factors in the application process relate to the observed outcomes for diversity research programs. There is some literature from graduate school, medical school, and undergraduate admissions that suggests a combination of cognitive, noncognitive, quantitative, and qualitative factors is an important component to target outcomes.

In a report from the GRE research board, 80 individuals were interviewed from 14 institutions and asked about their graduate school admissions process and the factors they associated with graduate student success (Walpole, Burton, Kanyi, & Jackenthal, 2002). There were key selection criteria found among those interviewed: GPA, GRE scores, letters of recommendation, and personal statements. Beyond this, the data were less uniform. However, some did agree that some qualitative measures were key to admission and success and these factors included motivation, curiosity, persistence, interpersonal skills, writing ability, integrity, commitment to field, creativity, leadership, and planning ahead (Walpole et al., 2002). Newer studies have shown that while quantitative measures like the GRE may be a predictor of first-semester grades in graduate school that neither grades nor GRE were a predictor of graduate school productivity nor GRE a predictor of passing qualifying exams, obtaining fellowships or time do degree (J. D. Hall, O’Connell, & Cook, 2017; Moneta-Koehler, Brown, Petrie, Evans, & Chalkley, 2017). In medical school admissions, several studies implicated the interview, where mostly noncognitive and qualitative factors are assessed, as a key element in student entry and success in the clinical components of medical school and beyond (Mercer & Puddey, 2011; Moruzi & Norman, 2009; Puryear & Lewis, 1981; Wagoner & Gray, 1979).

To make the most of qualitative and noncognitive factors, we think a rubric is necessary, but may be difficult to validate and train others for consistency. We use a very basic system where we score both qualitative and quantitative items on the application and interview. Research by Dawes, Faust, and Meehl (1989) promoted the value of this actuarial assessment versus merely clinical and argued that a numerical value can be given to any type of description of human interest (Dawes et al., 1989). Although we have not seen studies that have assessed their application process for diversity research programs, there are studies that have done actuarial assessments of the benefits of undergraduate research. Lopatto (2004, 2007) developed a survey instrument that measured the effects of undergraduate research and demonstrated that key benefits were experienced by URM students that included an increase in independence and positive influence on career plans (Lopatto, 2004, 2007). Thus, it is reasonable to use similar measures as we have done and as demonstrated above to evaluate the effectiveness of the application process for diversity research programs for correlation with outcomes.

We are not suggesting that our application is responsible for the increases in PhD outcomes, but improvement in this tool led to better selection, beyond the typical selection (e.g., high GPA, number of research experiences, or research with renowned investigators). We assume that our program components played a role. We made some changes where we made programming more consistent and intentional in the second cycle, for instance, we implemented mock interviews with faculty for both the summer and postbacc program and our graduate school prep course was taught in a similar way for both programs and by the same teacher for most of the second cycle. We are analyzing data from these components to see what themes were most prominent by those pursuing PhD. We also believe that research self-efficacy and self-identity is certainly important as shown by others (Maton et al., 2016; McGee & Keller, 2007). We plan to explore this avenue with our data as well as the gains made in areas that indicate research self-efficacy (e.g., increased competence in reading and interpreting literature, troubleshooting experiments, scientific communication, etc.) to see how these factor into our outcomes. We suggest that the success we have seen over the two cycles of the OGR program may be a part of a cumulative effect, where a combination of events led to higher PhD matriculation in Cycle 2 of our programs.

We propose that our revised application process captured applicants with high commitment to the PhD, and their previous or gained experiences in addition to their OGR experiences increased their self-confidence, research self-efficacy, and likely their science identity to pursue the PhD. This increased confidence along with their cumulative training experiences enhanced their profile and increased their admission to PhD programs.

### Limitations and Implications

At present, the size of our programs as well as the need for additional time to collect more data on the success of PhD matriculants remain limitations for quantitatively documenting the cumulative effects we propose above. It is clear that this is a small study, so results should be taken with caution. We emphasize that we are not implying that we have model outcomes as there are programs that have higher percentages of students entering PhDs, but we are celebrating our improvement and outlining how we got here. In light of this, our program has never focused on admitting students with the absolute highest quantitative measures or who were the strongest candidates for graduate school. Rather, we focused on grooming talent and, in latter years, identified applicants with more commitment. This underlines a unique measure and a limitation to our study: the lack of a control group. Ideally, we would have a comparison group to gauge whether our application process is truly capturing commitment and predicting entry. With such a group, we would compare our application process with similar programs but, for the results to matter, it would be necessary to control for factors such as GPA, school quality, and so on.

We also acknowledge that a potential limitation to our model of combining the summer and postbacc program data prevent us from distinguishing specific attributes of the two groups that may be contributing to their persistence to the PhD. For instance, we know why our participants pursued our postbacc program but we do not know why the undergraduates who pursued PhD immediately after baccalaureate did so. Our present study does not account for students who entered other postbaccs or other degrees prior to PhD either. There are a number of scenarios that can be imagined with these two groups that we did not test but may be interesting for future analysis, such as the entering characteristics of each group, and their paths to the PhD.

In this study, we only tested the number of proxies for PhD pursuit and the correlation of this number with the percentage of PhDs pursued to indicate that this was more than coincidental. A more comprehensive and conclusive study would involve the analysis of how these proxies were answered, how the proxies were evaluated, and if certain proxies were more predictive of PhD pursuit than others and testing the correlation of these responses with PhD pursuit and retention. We believe this type of thorough data analysis could lead to validation of the instrument we developed and would be an exciting venture, especially from the aspect of measuring noncognitive proxies for PhD commitment. Considering the drop in PhD matriculants in 2013, we think an exhaustive analysis could help us distinguish applicants who are on the PhD versus MD or other degree “fence.”

As mentioned earlier, there have been studies to develop a standardized tool to assess noncognitive factors as they relate to outcomes after completion of a program (Lopatto, 2004, 2007). But there have been some mixed results using instruments to measure noncognitive factors as predictors of student pursuits and success. Using the Non-Cognitive Questionnaire (NCQ), researchers found that graduation from college was predicted by noncognitive measures such as academic self-confidence and community service for both Black and White students, although stronger for Blacks (Tracey & Sedlacek, 1986). A study using a revised Non-Cognitive Questionnaire (NCQ-R) found that noncognitive measures, such as campus support and social integration, were significant in predicting undergraduate GPA for Black females, but not males (Hood-Ward, 1992). In contrast, a meta-analysis of over 9,000 undergraduates determined that the NCQ did not significantly predict GPA or persistence (Thomas, Kuncel, & Credé, 2007). These studies indicate the need for better tools and their limitations, but to our knowledge, no instruments currently exist that are directed at diversity research programs or graduate programs. We believe the development and validation of a “predictive application” would be of interest to diversity research programs and graduate programs as holistic review becomes more broadly accepted. This work may have the most implications for very small programs with few staff that must use their slots very wisely and have limited room for risk and little time to develop their program. As more best practices are revealed from researchers and model programs, we anticipate that funding agencies will focus more on what works rather than exploration and program development, thus programs may become more risk averse and will have limited time to develop and experience the ebbs and flows that exist. The foundation of this work and what could burgeon in the future may be of benefit to numerous diversity research programs. The application and selection process for programs and schools are not very transparent, mainly for reasons of competition. But formulating a process, including an application that better targets the desired outcomes of a program, can be both time and cost efficient and can improve chances of renewal for such programs or validate the existence of such programs with higher administrations at institutions while also contributing to the diversity of the future STEM workforce.

## Conclusion

We believe that we now have an application process that better selects students committed to the PhD path in the OGR program. We provide evidence for the first time that shows that the number of proxies for PhD pursuit in the application process for diversity research programs significantly correlates with the pursuit of degree outcomes aligned with the program’s mission. The research herein adds to the growing list of factors that are important in building a successful diversity research program that can inform leaders of such programs at the creation, design, and implementation stages. This study provides some foundational knowledge that can lead to validation of an instrument that will make the application process for diversity research programs more effective.

## Supplementary Material

Supplementary Material

## Figures and Tables

**Table 1.** Demographics for OGR Programs Disaggregated by Funding Cycle of Participation.

	Funding Cycle 1 2007–2011 (*n* = 49)		Funding Cycle 2 2012–2015 (*n* = 39)	
	*n*	%	*n*	%
Gender				
Male	27	55	18	46
Female	22	45	21	54
Ethnicity				
Hispanics	29	59	23	59
Non-Hispanics	20	41	15	39
No response	0	0	1	3
Race				
Asian	1	2	2	5
Black/African American	27	55	19	49
Caucasian	1	2	5	13
Other	0	0	3	8
Multiracial	1	2	3	8
Native American	1	2	0	0
No Response	18	37	7	18

*Note*. Percent columns may not add to 100% due to rounding to nearest whole percent. OGR = Opportunities in Genomics Research.

† Statistical significance, *p* ≤ .05.

**Table 2.** Degree Outcomes for OGR Programs Disaggregated by Funding Cycle of Participation.

	Funding Cycle 1 2007–2011 (*n* = 49)		Funding Cycle 2 2012–2015 (*n* = 39)	
Degree outcomes (All)				
Postbacc/master’s	4	8%	2	5%
STEM professional degree	11	22%	2	5%^†^
Other	21	43%	12	31%
PhD	13	27%	23	59%^†^
Degree outcomes (Blacks)				
Postbacc/master’s	3	11%	1	5%
STEM professional degree	6	22%	2	11%
Other	13	48%	5	26%
PhD	5	19%	11	58%^†^
Degree outcomes (Hispanics)				
Postbacc/master’s	0	0%	0	0%
STEM professional degree	5	25%	0	0%
Other	7	35%	5	33%
PhD	8	40%	10	67%

*Note*. Percent columns may not add to 100% due to rounding to nearest whole percent. OGR = Opportunities in Genomics Research; STEM = science, technology, engineering, and math.

† Statistical significance, *p* ≤ .05.

**Table 3.** Undergraduate GPAs for OGR Programs Disaggregated by Funding Cycle of Participation.

					95% confidence interval for *M*			
	*n*	*M*	*SD*	*SE*	Lower bound	Upper bound	Minimum	Maximum
GPA								
Funding Cycle 1 2007–2011	49	3.45	0.34	0.05	3.35	3.55	2.50	4.00
Funding Cycle 2 2012–2015	39	3.41	0.29	0.05	3.32	3.51	2.98	4.02
STEM GPA								
Funding Cycle 1 2007–2011	49	3.29	0.48	0.07	3.16	3.43	2.00	4.00
Funding Cycle 2 2012–2015	39	3.32	0.37	0.06	3.20	3.44	2.46	4.05

*Note*. GPA = grade point average; OGR = Opportunities in Genomics Research; STEM = science, technology, engineering, and math.

**Table 4.** Institution Classifications for OGR Applicants Disaggregated by Funding Cycle of Participation, Then Filtered for PhD.

	Funding Cycle 1 2007–2011 (*n* = 49) (All)		Funding Cycle 2 2012–2015 (*n* = 39) (All)		Funding Cycle 1 2007–2011 (n = 13) (PhD)		Funding Cycle 2 2012–2015 (*n* = 23) (PhD)	
	*n*	%	*n*	%	*n*	%	*n*	%
MSI classification								
Non-MSI	15	31	20	51	3	23	12	52
HBCU	18	37	9	23	4	31	5	22
HSI	16	33	10	26	6	46	6	26
Carnegie Classification								
Associates	1	2	0	0	0	0	0	0
Baccalaureate	12	25	9	23	3	23	4	17
Master’s	8	16	9	23	1	8	6	26
Doctoral	6	12	7	18	1	8	6	26
Research	22	45	14	36	8	62	7	30

*Note*. Percent columns may not add to 100% due to rounding to nearest whole percent. OGR = Opportunities in Genomics Research; MSI = minority-serving institution; HBCU = historically Black colleges and university; HSI = Hispanic serving institutions.

**Table 5.** Mean Rankings of Graduate School Programs, for All, Black, and Hispanic OGR Program Participants, Disaggregated by Funding Cycle of Participation.

					95% confidence interval for *M*			
	*n*	*M*	*SD*	*SE*	Lower bound	Upper bound	Minimum	Maximum
All								
Funding Cycle 1 2007–2011	11	44	38.6	11.6	17.7	69.58	1	115
Funding Cycle 2 2012–2015	22	29	30.3	6.5	15.5	42.36	5	139
Black								
Funding Cycle 1 2007–2011	4	58	42.9	21.5	−10.6	126.07	11	115
Funding Cycle 2 2012–2015	11	20^†^	12.7	3.8	11.7	28.71	5	42
Hispanic								
Funding Cycle 1 2007–2011	7	36	36.8	13.9	1.5	69.6	1	93
Funding Cycle 2 2012–2015	9	37	44.1	14.7	3.1	70.9	9	139

*Note*. OGR = Opportunities in Genomics Research; *n* = number of participants who attended ranked graduate programs.

† Statistical significance, *p* ≤ .05.

**Table 6.** OGR Students Accepted to WU PhD Program, Disaggregated by Funding Cycle of Participation.

	Funding Cycle 1 2007–2011 (*n* = 49)		Funding Cycle 2 2012–2015 (*n* = 39)	
	*n*	%	*n*	%
Accepted to WU PhD				
No	47	96	28	72
Yes	2	4	11	28^†^

*Note*. OGR = Opportunities in Genomics Research; WU = Washington University.

† Statistical significance, *p* ≤ .05.
