# Supplementary material for: Proxies for Success: How the Application Process Correlates to PhD Pursuit for a Small Diversity Research Program
Source: Sage Open. Author manuscript; Available in PMC 2024 Jun 6. (PMC11156220; doi:10.1177/2158244017727040)
Supplement: Supplementary Material [file NIHMS1966728-supplement-Supplementary_Material.pdf]

## Supplemental Material - Comparison of application proxies for 2008 and 2015

### Application form

| Sample Proxies in 2008 | Sample Proxies in 2015 |
|---|---|
| **Closed-ended** | |
| • Do you plan to enter a graduate/professional program after graduation? (Yes/No)<br>• Indicate your graduate/professional program of interest: PhD, MD/PhD, MD, DDS, Masters, Other<br>• Have you ever taken the GRE? Yes/No | Indicate your graduate/ professional program of interest: PhD, MD/PhD, MD, DDS, Masters, Other<br><br>Select all that apply to the indicated questions<br>__I plan to enter a graduate program after graduation?<br>__I plan to enter a professional (e.g. medical, dental etc) program after graduation<br>__I have applied to for admission to graduate programs<br>__I have applied for admission to professional programs<br>__I have interests other than graduate or professional school<br><br>• Do you plan to take any of the following? (check all that apply)<br>• GRE , MCAT, DAT, PCAT, LSAT, GMAT, Other (indicate)<br>• Have you ever taken the GRE? Yes/No<br>• If yes, indicate date and scores<br>• Have you ever taken the MCAT? Yes/No<br>• If yes, indicate your last scores?<br>• Have you participated in any research excluding course work?<br>• Have you or do you currently participate in any of the following programs: MARC USTAR, LSAMP, Leadership Alliance, NSF REU, etc.<br><br>**Indicate if you have: (yes/no)**<br>• Attended a conference or societal meeting for students majoring in the biomedical sciences (e.g., ABRCMS, SACNAS)<br>• Presented a poster at a biomedical conference or societal meeting<br>• Participated in sessions focused on graduate school planning and preparation for the biomedical field<br>• Participated in a business or industry related internship related to a biomedical field<br>• Participated in a GRE/MCAT preparation course or workshop<br>• Prepared a science abstract for your research project<br>• Served as a mentor/tutor to other undergraduates majoring in biomedical areas<br>• Co-authored a biomedical research publication (for a peer reviewed journal) |
| **Open-Ended** | |
| • List any previous research experience and title of research project, etc<br>• Write a statement indicating the following: your interest in participating, your career and educational goals and how previous research experience and your overall interest in science have influenced these goals. | • Who or what has influenced your potential career choice?<br>• What are your current research or science interests and why?<br>• Why do you want to pursue a career in science, technology, engineering or mathematics?<br>• What steps have you taken to pursue your career of interest?<br>• Why do you want to participate in the OGR program?<br>• Provide evidence of published abstracts<br>• Provide evidence of published manuscripts |

### Faculty recommendation form

| Sample Proxies in 2008 | Sample Proxies in 2015 |
|---|---|
| **Closed-ended** | |
| Rate the applicant (outstanding to below average)<br>• Scientific knowledge<br>• Communicating scientific findings orally<br>• Communicating scientific findings in writing (lab report, abstract)<br>• Reading and interpreting scientific literature | Rate the applicant (outstanding to below average)<br>• Scientific knowledge<br>• Communicating scientific findings orally<br>• Communicating scientific findings in writing (lab report, abstract)<br>• Reading and interpreting scientific literature<br><br>Indicate the program which the candidate is best suited (e.g., PhD, MD, and others) |
| **Open-Ended** | |
| • None | • None |

**Phone Interview Protocol**

| Sample Proxies in 2008 | Sample Proxies in 2015 |
|---|---|
| **Closed-ended** | |
| • None | • None |
| **Open-Ended** | |
| • Do you enjoy the labs associated with your courses?<br>• Have you had a summer research experience?<br>• How much exposure or experience do you have reading scientific articles?<br>• Why is it important to you to pursue a research opportunity?<br>• What do you expect to learn in the lab?<br>• What do you want to get from this program in general?<br>• What are your main career interests (ES) | • Tell me about you, your interests? 1-2 min<br>• Do you enjoy the labs associated with your courses? Why or why not?<br>• What specifically do you like or dislike? **(for younger/inexperienced students)?**<br>• Have you had a summer/academic year research experience or describe your research experience?<br>• Do you enjoy research?<br>• What do you enjoy most?<br>• How much exposure or experience do you have reading scientific articles?<br>• How often do you read articles?<br>• What is the most pressing issue in science today?<br>• What do you think has been the most important discovery?<br>• Describe a day in the life of a scientist/researcher?<br>• When you think of a scientist or researcher, what adjectives would you use to describe this person? (ES)<br>• Name some of the careers are available to people a PhD in STEM?<br>• Why did you major in (fill in blank)?<br>• Why is it important to you to pursue an internship with OGR?<br>• What do you expect to learn in the lab?<br>• What do expect to learn from the program in general?<br>• What degree do you plan to pursue after completing undergraduate? Why?<br>• Where do you see yourself in 5 years? 10 years? |
